# Efficacy and safety of tigecycline versus levofloxacin for community-acquired pneumonia

**DOI:** 10.1186/1471-2466-9-44

**Published:** 2009-09-09

**Authors:** Cristina Tanaseanu, Slobodan Milutinovic, Petre I Calistru, Janos Strausz, Marius Zolubas, Valeriy Chernyak, Nathalie Dartois, Nathalie Castaing, Hassan Gandjini, C Angel Cooper

**Affiliations:** 1 St. Pantelimon Clinical Emergency Hospital, Bucharest, Romania; 2 General Hospital Sveti Duh, Zagreb, Croatia; 3 Clinic of Infectious and Tropical Diseases "Dr. Victor Babes" Bucharest, Romania; 4 Pulmonology Hospital, Torokbalint, Hungary; 5 Klaipeda Regional Hospital, Klaipeda, Lithuania; 6 Cherkassy Regional Hospital, Cherkassy, Ukraine; 7 Wyeth Research, Paris, France; 8 Wyeth Research, Collegeville, PA, USA

## Abstract

**Background:**

Tigecycline, an expanded broad-spectrum glycylcycline, exhibits in vitro activity against many common pathogens associated with community-acquired pneumonia (CAP), as well as penetration into lung tissues that suggests effectiveness in hospitalized CAP patients. The aim of the present study was to compare the efficacy and safety of intravenous (IV) tigecycline with IV levofloxacin in hospitalized adults with CAP.

**Methods:**

In this prospective, double-blind, non-inferiority phase 3 trial, eligible patients with a clinical diagnosis of CAP supported by radiographic evidence were stratified by Fine Pneumonia Severity Index and randomized to tigecycline or levofloxacin for 7-14 days of therapy. Co-primary efficacy endpoints were clinical response in the clinically evaluable (CE) and clinical modified intent-to-treat (c-mITT) populations at test-of-cure (Day 10-21 post-therapy).

**Results:**

Of the 428 patients who received at least one dose of study drug, 79% had CAP of mild-moderate severity according to their Fine score. Clinical cure rates for the CE population were 88.9% for tigecycline and 85.3% for levofloxacin. Corresponding c-mITT population rates were 83.7% and 81.5%, respectively. Eradication rates for *Streptococcus pneumoniae *were 92% for tigecycline and 89% for levofloxacin. Nausea, vomiting, and diarrhoea were the most frequently reported adverse events. Rates of premature discontinuation of study drug or study withdrawal because of any adverse event were similar for both study drugs.

**Conclusion:**

These findings suggest that IV tigecycline is non-inferior to IV levofloxacin and is generally well-tolerated in the treatment of hospitalized adults with CAP.

**Trial registration:**

NCT00081575

## Background

Community-acquired pneumonia (CAP) occurs in approximately one to five per 1000 of the adult population per year [1-3] and is associated with rates of mortality ranging from a low of 5% up to 30%-50% for patients with multiple comorbidities and requiring intensive care [2,4,5]. *Streptococcus pneumoniae *(the most common etiologic agent), nontypeable *Haemophilus influenzae*, and the atypical organisms (i.e., *Mycoplasma pneumoniae*, *Chlamydia pneumoniae*, and *Legionella pneumophila*) are the most frequently isolated pathogens from patients of any age who require hospitalization for CAP [2,5-10]. Rates of multidrug-resistant *S. pneumoniae *have been reported to be >30% worldwide and *H. influenzae *beta-lactamase production varies by country, ranging from 12% to 27% [11-14].

Initial antimicrobial treatment for patients with CAP should provide appropriate coverage against the most common causative organisms, including resistant strains, while evaluating whether monotherapy or combination therapy is required. For hospitalized patients who do not require admission to the intensive care unit (ICU), the most recent guidelines published by several authorities recommend the combination of an extended-spectrum cephalosporin or beta-lactam/beta-lactamase inhibitor combination with the option of adding a macrolide or monotherapy with a newer fluoroquinolone [5,7-10].

Tigecycline, a first-in-class, expanded broad-spectrum glycylcycline, demonstrates *in vitro *activity against many commonly encountered respiratory bacteria, including multiple resistant gram-positive, gram-negative, anaerobic, and "atypical" bacteria such as multidrug-resistant *S. pneumoniae *and beta-lactamase producing *H. influenzae *[15]. In this first phase 3 study in CAP with tigecycline, the primary objective was to compare the efficacy and safety of intravenous (IV) tigecycline with IV levofloxacin in the treatment of patients with CAP requiring hospitalization. Levofloxacin was chosen as the comparator because it is commonly prescribed worldwide and it is recommended in guidelines for the treatment of CAP [5,8-10].

## Methods

### Study design and patient enrolment criteria

A prospective, double-blind (third-party unblinded), randomized, multicentre, phase 3 study was conducted from January 2004 to January 2005 at 62 centres in 20 countries in Europe, Africa, and the Asia Pacific region. Approximately 400 subjects were enrolled to obtain 240 clinically evaluable subjects and to ensure with 90% probability that the lower bound of a two-sided 95% confidence interval for the true difference in efficacy would not be less than -15%. The protocol was reviewed and approved by each investigator's independent ethics committee or institutional review board in accordance with local regulations and good clinical practices. Written informed consent was to be obtained from each patient or his or her guardian before commencement of any study-specific procedure.

Subjects were stratified at randomization by the Fine Pneumonia Severity Index score (V, IV, III, or less than III) [16]. In a post-hoc analysis, the severity of each subject's pneumonia was also categorized using an estimate of the CURB-65 prediction severity tool, using a notation of "altered mental status" under medical history for "confusion" [17]. Subjects were assigned in a 1:1 ratio to treatment using a computerized system of automated telephone randomization, to receive either IV tigecycline (initial 100-mg dose given by infusion over a 60-minute period, followed by 50 mg IV every 12 hours) or levofloxacin (500 mg once-daily or twice-daily based on investigator's discretion, administered over a 60-minute period; or for subjects with creatinine clearance 20-49 mL/min, an initial 500-mg dose followed by 250 mg once- or twice-daily) for at least 7 days, unless a clinical failure, and up to a maximum of 14 days. An unblinded third party prepared the masked test articles and an unblinded nurse administered the test article. The person(s) responsible for preparing and/or administering the test article were not involved in the assessment or evaluation of the subject for safety or efficacy. Each subject's blinded data set was reviewed to ensure the accuracy and integrity of the data.

### Patient population

Male or non-pregnant/non-lactating female patients ≥18 years of age (in Bulgaria only from 18 to ≤ 70 years of age due to local requirement) hospitalized with clinical signs and symptoms of CAP who required initial parenteral therapy for at least 7 days were considered for enrolment. Each patient was to have fever within 24 hours of randomization (oral temperature >38°C/100.4°F, axillary temperature >38.1°C/100.6°F, tympanic membrane temperature >38.5°C/101.2°F, or a rectal/core temperature ≥ 39°C/102.2°F) or hypothermia (core temperature <35°C/95°F). Each patient also was to have at least two of the following signs and symptoms consistent with CAP: cough with production of purulent or mucopurulent sputum; auscultatory findings on pulmonary examination suggestive of pulmonary consolidation (dullness to percussion, rales/rhonchi, or bronchial breath sounds); dyspnoea or tachypnoea; white blood cell (WBC) count >10,000/mm^3^, or >15% immature neutrophils (bands), and/or leucopoenia with a total WBC count <4500/mm^3^; and hypoxemia (PO_2 _< 60 mm Hg or oxygen saturation <90% while the subject was breathing room air). Radiologically-confirmed evidence of a new or progressive infiltrate(s) consistent with bacterial pneumonia within 48 hours before receiving the first dose of study drug was also mandatory.

Key exclusion criteria included: hospitalization within 14 days before the onset of symptoms; residence in a long-term care facility ≥14 days before the onset of symptoms; sustained shock or required treatment in an intensive care unit; known or suspected concomitant bacterial infection requiring treatment with an additional systemic antibacterial agent; received more than one dose of systemic antibacterial therapy (or received a once-daily antibiotic) to treat this episode of CAP prior to receiving the first dose of study drug, unless a clinical failure; and known or suspected *Pseudomonas*, *Pneumocystis carinii*, *Legionella *pneumonia, or tuberculosis infection.

### Clinical evaluations

Eligible patients were evaluated, and clinical signs and symptoms recorded at serial visits: baseline (within 24 hours of first study drug dose), during treatment, early follow-up (Day 2-4 post-therapy), and test-of-cure (Day 10-21 post-therapy). Pulse oximetry and/or arterial blood gases were obtained at baseline, end of therapy, early follow-up, and at the test-of-cure visits. Chest x-rays were obtained at baseline (within 48 hours of receiving first dose of study drug) and were repeated at the test-of-cure visit. Clinical responses were graded as cure, failure, or indeterminate at the test-of-cure assessment.

### Microbiologic evaluations

Sputum samples, when available, were collected prior to initiation of study drug therapy and submitted to a local laboratory for Gram staining, culture, and susceptibility testing. Specimens obtained by deep expectoration or nasotracheal aspiration were considered appropriate for culture if the Gram stain revealed <10 squamous epithelial cells and >25 leukocytes/low-power field. Blood specimens (2 sets at least 15 minutes apart from 2 different sites) were to be drawn for culture at enrolment, and if positive were to be repeated at day 3 and at the discretion of the investigator until confirmed negative, or if the subject was a treatment failure. Urine specimens were collected at baseline for *Legionella *and pneumococcal urinary antigens. Serology for *Chlamydia*, *Mycoplasma*, and *Legionella *was to be obtained for all patients at baseline and 6 ± 2 weeks after the baseline collection of blood samples.

All aerobic and anaerobic bacterial isolates, regardless of the source of cultured material, were to be identified and tested at the investigator's laboratory and then confirmed at a central laboratory (Covance Central Laboratory Services, Inc., Geneva, Switzerland) according to standard procedures.

### Safety and tolerability assessments

Each patient who received at least one dose of study drug was evaluated for safety (modified intent-to-treat [mITT] population) based on medical history and physical examinations, reports of clinical adverse events (AEs), and findings from 12-lead electrocardiograms (ECGs) and serum chemistry, haematology, and coagulation tests. Adverse events were to be recorded throughout the study period, up to and including the test-of-cure visit (or 14 days after the last dose of study drug, whichever was greater). Before unblinding, the investigator categorized the severity of each adverse event and the potential for relationship to the study drug. Severity of nausea and vomiting was categorized according to the National Cancer Institute Common Toxicity Criteria: grade 1 (mild), 2 (moderate), 3 (severe), and 4 (life-threatening). Serious adverse events (i.e., those that were life-threatening, led to prolongation of the existing hospitalization, or caused persistent or significant disability or incapacity, or death) were also recorded.

### Statistical analysis

The primary efficacy endpoint of the study was the clinical response at the test-of-cure visit (10-21 days after therapy) for the co-primary clinically evaluable (CE) and clinical modified ITT (c-mITT) populations. Secondary analyses included clinical response rates in the microbiologically evaluable (ME) and microbiologic modified ITT (m-mITT) populations, monomicrobial versus polymicrobial infections, and by isolate, as well as microbiologic response at the test-of-cure visit by patient and isolate.

Statistical analysis was performed by the Clinical Biostatistics Department of Wyeth Research, Collegeville, PA. Categorical baseline demographic and medical variables were analyzed using the Fisher exact test. Continuous variables were compared using a one-way analysis of variance (ANOVA) model with treatment as a factor. Between-group comparisons of adverse events were analyzed by using the Fisher exact test. For laboratory tests, vital signs, and ECG results, within-group changes from baseline were analyzed by using a paired t-test and between-group comparisons were made by using the analysis of covariance, adjusting for baseline value. The difference between treatment groups in the percentage of premature discontinuation from study drug was evaluated by using a two-sided Fisher exact test.

Non-inferiority of tigecycline compared with levofloxacin was evaluated for clinical response by using a two-sided 95% confidence interval for the true difference in efficacy (tigecycline minus levofloxacin) adjusted for the stratification variable (Fine Pneumonia Severity Index score) used at the time of randomization. Non-inferiority was concluded if the lower limit of the two-sided 95% CI was greater than or equal to -15%. For all subpopulation analyses (e.g., monomicrobial versus polymicrobial infection), an adjusted difference between treatment groups with its 95% CI was calculated from a generalized linear model with a binomial probability function and an identity link (Proc GENMOD).

## Results

**Figure 1:**
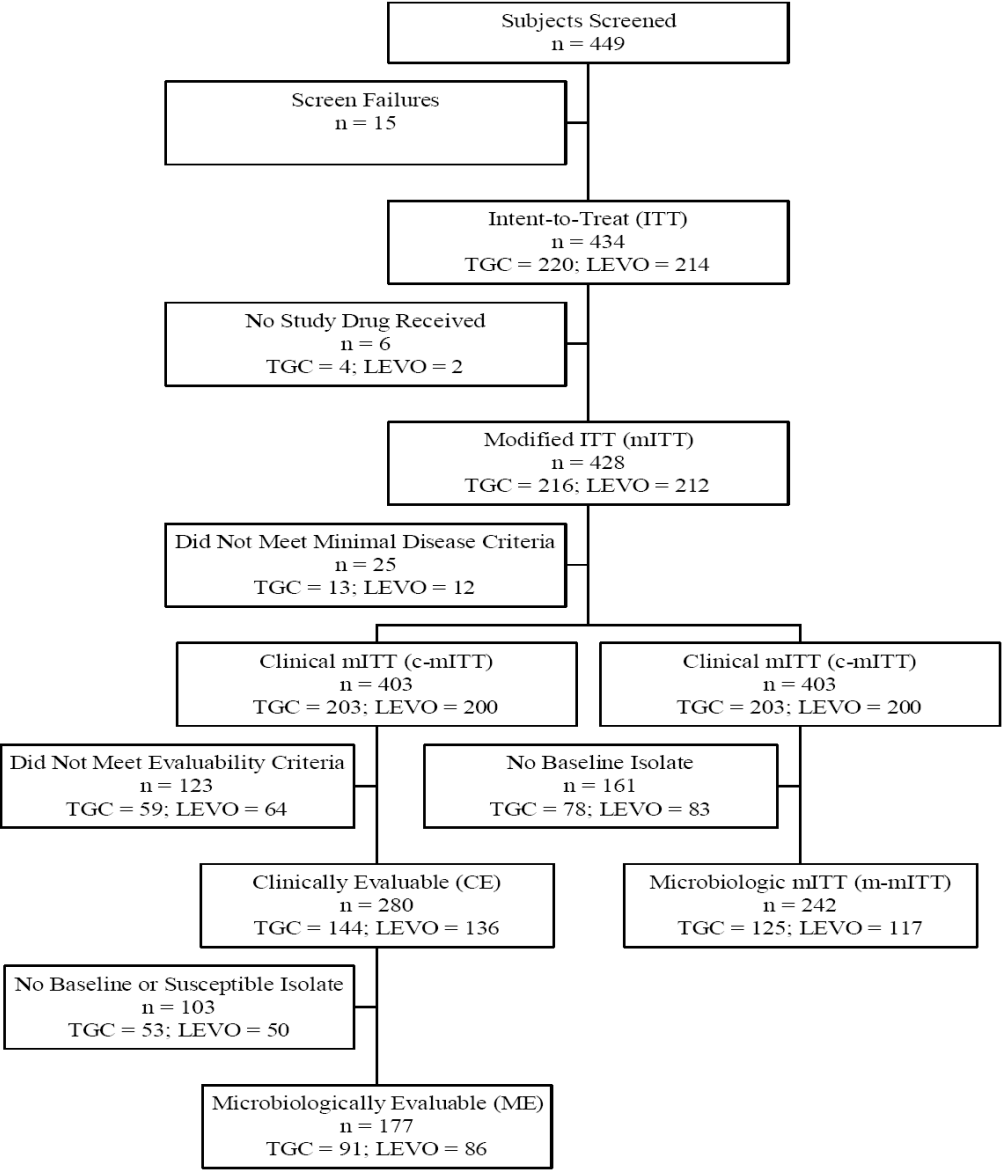
**Flow diagram: disposition of patients**. Patients randomized were included in the intent-to-treat (ITT) population. Those who received at least one dose of study drug comprised the modified ITT (mITT) population, and patients in the mITT population who had clinical evidence of CAP by meeting the minimal disease criteria made up the clinical modified ITT (c-mITT) population. The microbiologic modified intent-to-treat (m-mITT) population consisted of c-mITT subjects who had 1 or more baseline isolates identified. Patients in the c-mITT population were considered to be clinically evaluable (CE) if they satisfied inclusion and exclusion criteria, received no more than one dose of a non-once daily non-study antibacterial agent (single agent or combination therapy) to treat the current episode of CAP before the first dose of study drug, did not receive other concomitant systemic antimicrobial therapy unless a treatment failure, received at least 2 full days of study drug if clinical failure or 5 full days of study drug if clinical cure, were adherent with therapy (i.e., ≥ 80% but ≤ 120% of medication administered), had an assessment of cure or failure at the test-of-cure visit (10-21 days after the last dose of therapy), and the study blind was maintained. The microbiologically evaluable (ME) population included CE patients for whom at least one isolate was identified from the baseline culture that was susceptible to both test drugs and who had a microbiologic response that could be classified as eradication, persistence, or superinfection at the test-of-cure visit.

A total of 449 patients were screened for study participation, of which 434 were randomly assigned to receive tigecycline or levofloxacin, constituting the intent-to-treat (ITT) population, and 428 received at least 1 dose of study medication, constituting the modified ITT (mITT) population (Figure 1).

### Demographics and baseline medical characteristics

Demographic and baseline medical characteristics for the mITT population are summarized in Table 1 and were similar for the two treatment groups, with a mean age of 49.8 ± 17.7 years and a slight predominance of male enrolment (62%). All subjects required hospitalization although most subjects were considered to have mild-moderate CAP (79% with Fine score I-III). Demographic and baseline medical characteristics in the CE population displayed similar results (Table 1).

**Table 1:** Baseline Demographic and Medical Characteristics*

	**Study Population**			
	
	**mITT**		**CE**	
	
**Characteristic**	**Tigecycline** **(n = 216)**	**Levofloxacin** **(n = 212)**	**Tigecycline** **(n = 144)**	**Levofloxacin** **(n = 136)**
Male, n (%)	131 (61)	133 (63)	91 (63)	87 (64)
Race, n (%)				
White	189 (88)	184 (87)	129 (90)	117 (86)
Black	2 (<1)	4 (2)	-	3 (2)
Asian	5 (2)	7 (3)	3 (2)	7 (5)
Other	20 (9)	17 (8)	12 (8)	9 (7)
Mean age ± SD, years (range)	49.9 ± 18.1 (17--92)	49.7 ± 17.4 (18--85)	52.8 ± 16.9 (18--89)	50.4 ± 17.0 (18--85)
Fine Pneumonia Severity Score Index, n (%)				
I	32 (15)	41 (19)	18 (13)	23 (17)
II	79 (37)	61 (29)	49 (34)	40 (29)
III	59 (27)	66 (31)	40 (28)	42 (31)
IV	44 (20)	42 (20)	35 (24)	30 (22)
V	2 (<1)	2 (<1)	2 (1)	1 (<1)
Prior antibiotic failure, n (%)	44 (20)	55 (26)	9 (6)	15 (11)
Presence of underlying medical conditions				
COPD, n (%)	14 (7)	19 (9)	10 (7)	14 (10)
Diabetes mellitus, n (%)	24 (11)	25 (12)	17 (12)	16 (12)
Alcohol abuse, n (%)	14 (7)	10 (5)	10 (7)	7 (5)
Neoplastic disease, n (%)	-	2 (<1)	-	2 (2)
Liver disease, n (%)	15 (7)	8 (4)	10 (7)	4 (3)
Congestive heart disease, n (%)	15 (7)	15 (7)	13 (9)	10 (7)
Cerebrovascular disease, n (%)	12 (6)	17 (8)	8 (6)	13 (10)

*There were no statistically significant differences between the treatment groups for demographic or baseline medical characteristics for both the mITT and CE populations.

### Clinical efficacy

The clinical efficacy of tigecycline was found to be non-inferior (*P *< 0.001) to that of levofloxacin in the CE population, with clinical cure rates of 88.9% for tigecycline versus 85.3% for levofloxacin (adjusted difference of 3.6, 95% CI -4.5, 11.8; *P *= 0.4025; Table 2). Analysis of clinical response at the test-of-cure assessment for the CE population by Fine score category also showed the non-inferiority of tigecycline compared with levofloxacin. Similar findings were observed for clinical response using estimated CURB-65 score distributions (Table 2). Clinical cure rates for patients with diabetes were 100% (17/17) for tigecycline- vs. 75% (12/16) for levofloxacin-treated patients. Similar cure rates were reported for subjects with impaired renal function (i.e., creatinine clearance ≤70 mL/min): 91.3% (42/46) for tigecycline and 78.3% (36/46) for levofloxacin. Noninferiority of tigecycline was also demonstrated in the c-mITT population (*P *< 0.001), with clinical cure rates of 83.7% for tigecycline versus 81.5% for levofloxacin (adjusted difference of 2.0, 95% CI -5.5, 9.6; *P *< 0.6269). For both the ME and m-mITT populations, tigecycline was also efficacious and statistically noninferior to levofloxacin. When analyzed by the type of infection (polymicrobial and monomicrobial) for the ME and m-mITT populations, the results were generally similar within each of the treatment groups, and no significant differences in cure rates were observed between the two treatment groups (Table 3).

**Table 2:** Cure Rates at the Test-of-Cure Visit*

	**Tigecycline**		**Levofloxacin**		**Difference (Tigecycline - Levofloxacin)**		
	**n/N**	**% (95% CI)**	**n/N**	**% (95% CI)**	**% (95% CI)**	**Test for Non-inferiority** **p-Value**	**Test for Difference** **p-Value**

CE, Overall	128/144	88.9 (82.6,93.5)	116/136	85.3 (78.2, 90.8)	3.6 (-4.5, 11.8)	<0.001	0.4025
Fine <III	60/67	89.6 (79.7, 95.7)	55/63	87.3 (76.5, 94.4)	2.3 (-10.3, 14.8)	0.0026	0.8993
Fine III	34/40	85.0 (70.2, 94.3)	35/42	83.3 (68.6, 93.0)	1.7 (-16.6, 19.9)	0.0388	1.0000
Fine IV	32/35	91.4 (76.9, 98.2)	25/30	83.3 (65.3, 94.4)	8.1 (-11.2, 27.4)	0.0079	0.5463
Fine V	2/2	100.0 (15.8, 100.0)	1/1	100.0 (2.5, 100.0)	0 (-75.0, 75.0)	-	-
Estimated CURB-65 0-1	87/97	89.7 (81.9, 94.9)	79/93	84.9 (76.0, 91.5)	4.7 (-5.6, 15.3)		
Estimated CURB-65 2	31/36	86.1 (70.5, 95.3)	30/34	88.2 (72.5, 96.7)	-2.1 (-20.3, 16.6)		
CURB-65 ≥ 3	10/11	90.9 (58.7, 99.8)	7/9	77.8 (40.0, 97.2)	13.1 (-25.3, 51.7)		

c-mITT	170/203	83.7 (77.9, 88.5)	163/200	81.5 (75.4, 86.6)	2.0 (-5.5, 9.6)	<0.001	0.6269

*At the test-of-cure visit, each patient's response was categorized as one of the following: *Cure*---All signs and symptoms of pneumonia present at baseline were improved or resolved at test-of-cure with no worsening or appearance of new signs and symptoms of pneumonia and no requirement for further antibiotic therapy. Chest radiographs were improved or not worse. *Failure*---Persistence or worsening in signs and symptoms of the acute process with either failure to show improvement in the clinical findings, initial improvement in signs and symptoms followed by clinically significant worsening before the test-of-cure assessment, additional antimicrobial therapy required, progression of chest radiograph abnormalities, or death after study day 2 because of pneumonia; or *Indeterminate*---the patient was lost to follow-up, or died within 2 days after the first dose of study drug for any reason, or died after 2 days because of noninfectious-related reasons or infection other than pneumonia (as judged by the investigator). CE = clinically evaluable population; c-mITT = clinical-modified intent-to-treat population.

**Table 3:** Cure Rates by Monomicrobial/Polymicrobial Infection at the Test-of-Cure Visit

	**Tigecycline**		**Levofloxacin**		**Difference (Tigecycline -- Levofloxacin)**
	**n/N**	**% (95% CI)**	**n/N**	**% (95% CI)**	**% (95% CI)**

ME					
Monomicrobial	53/58	91.4 (81.0, 97.1)	56/64	87.5 (76.8, 94.4)	3.9 (-9.0, 16.3)
Polymicrobial	25/28	89.3 (71.8, 97.7)	20/21	95.2 (76.2, 99.9)	-6.0 (-25.1, 16.6)
m-mITT					
Monomicrobial	74/84	88.1 (79.2, 94.1)	76/88	86.4 (77.4, 92.8)	1.7 (-9.4, 12.7)
Polymicrobial	28/34	82.4 (65.5, 93.2)	23/25	92.0 (74.0, 99.0)	-9.6 (-28.4, 12.4)

ME = microbiologically evaluable population; m-mITT = microbiologic-modified intent-to-treat population.

In the tigecycline treatment group, the clinical cure rate for patients with bacteremia (ME population, 83.3%; 10/12) was similar to those who did not have bacteremia (91.9%; 68/74) at baseline. Corresponding rates for the levofloxacin group were 60% (3/5) and 91.3% (73/80), respectively. Most of the cases of bacteremia were caused by *S. pneumoniae*. In the tigecycline treatment group, the cure rate for subjects with *S. pneumoniae *bacteremia (ME population) was 90.9% (10/11), which was similar to those who did not have bacteremia (90.7%; 68/75). Corresponding rates for the levofloxacin group were 60% (3/5) and 91.3% (73/80), respectively.

### Microbiologic efficacy

Eradication rates (generally presumed, based on clinical response) at the test-of-cure visit for common respiratory pathogens (defined by respiratory culture, urinary antigen, or serology) within the ME population were similar between the two treatment groups (Table 4). For *S. pneumoniae*, the most common isolate, eradication rates were similar for tigecycline (92%) and levofloxacin (89%). Both study drugs eradicated 100% of penicillin-intermediate (n = 8) and penicillin-resistant (n = 5) strains. *M. pneumoniae*, the most commonly identified atypical organism, was eradicated in 96% (24/25) of tigecycline- and 92% (22/24) of levofloxacin-treated patients. There were no obvious differences in the eradication rates of other organisms, albeit the number of isolates was generally small.

**Table 4:** Microbiologic Response for Common Respiratory Pathogens at Test-of-Cure Visit in the ME Population

	**Tigecycline**			**Levofloxacin**		
**Isolate**	**n/N**	**%**	**(95% CI)**	**n/N**	**%**	**(95% CI)**
*Streptococcus pneumoniae*	46/50	92.0	(80.8, 97.8)	32/36	88.9	(73.9, 96.9)
Penicillin-intermediate *S. pneumoniae*	3/3	100	(29.2,100)	5/5	100	(47.8, 100)
Penicillin-resistant *S. pneumoniae*	2/2	100	(15.8, 100)	3/3	100	(29.2, 100)
*Haemophilus influenzae*	8/11	72.7	(39.0, 94.0)	6/7	85.7	(42.1, 99.6)
*Haemophilus parainfluenzae*	5/5	100	(47.8, 100)	9/9	100	(66.4, 100)
*Klebsiella pneumoniae*	4/4	100	(39.8, 100)	7/7	100	(59.0, 100)
*Staphylococcus aureus *(all non-MRSA)	7/9	77.8	(40.0, 97.2)	6/6	100	(54.1, 100)
*Chlamydia pneumoniae*	5/5	100	(47.8, 100)	11/11	100	(71.5, 100)
*Mycoplasma pneumoniae*	24/25	96.0	(79.6, 99.9)	22/24	91.7	(73.0, 99.0)
*Legionella pneumophila*	3/3	100	(29.2, 100)	5/5	100	(47.8, 100)

MRSA: methicillin-resistant *Staphylococcus aureus*

### Susceptibility data

Pretherapy *in vitro *activity against baseline isolates for tigecycline and levofloxacin for the ME population are outlined in Table 5. The MIC_90 _for tigecycline against the common respiratory pathogens was ≤1.0 μg/mL. Specifically, the mean MIC_90 _for tigecycline against *S. pneumoniae *(all isolates) was 0.06 μg/mL. No patients were identified with decreased susceptibility to tigecycline.

**Table 5:** MIC Range, and MIC_50 _and MIC_90_Values of Common Respiratory Pathogens in the ME Population

	**Tigecycline**				**Levofloxacin**			
**Isolate**	**n**	**MIC Range**	**MIC**_50_	**MIC**_90_	**n**	**MIC Range**	**MIC**_50_	**MIC**_90_
*Streptococcus pneumoniae* (penicillin susceptible)	45	0.030, 0.120	0.060	0.060	45	0.250, 2.000	1.000	1.000
Penicillin-intermediate *S. pneumoniae*	8	0.060, 0.060	NA	NA	8	1.000, 1.000	NA	NA
Penicillin-resistant *S. pneumoniae*	5	0.060, 0.060	NA	NA	5	1.000, 2.000	NA	NA
*Haemophilus influenzae*	18	0.120, 0.500	0.250	0.500	18	0.120, 0.120	0.120	0.120
*Haemophilus parainfluenzae*	13	0.250, 0.500	0.500	0.500	13	0.120, 0.120	0.120	0.120
*Klebsiella pneumoniae*	11	0.250, 2.000	0.500	1.000	11	0.120, 0.500	0.120	0.120
*Staphylococcus aureus *(all non-MRSA)	15	0.120, 0.250	0.120	0.120	15	0.120, 0.250	0.120	0.250

MRSA: methicillin-resistant *Staphylococcus aureus*

### Safety and tolerability

Regardless of study drug causality or severity, the frequency and distribution of treatment-emergent adverse events (TEAEs) occurring in at least 3% of patients in either treatment group is outlined in Table 6. Significantly more tigecycline-treated patients (135; 62.5%) reported one or more TEAEs compared with levofloxacin (100; 47.2%) (*P *= 0.002). Significantly more patients in the tigecycline group reported nausea (26.9% tigecycline versus 8.5% levofloxacin, *P *< 0.001), vomiting (16.7% tigecycline versus 6.6% levofloxacin, *P *= 0.001), and leukocytosis (6.9% tigecycline versus 0.9% levofloxacin, *P *= 0.002), while hypokalaemia was reported significantly more frequently in the levofloxacin group (0.5% tigecycline versus 3.8% levofloxacin, *P *= 0.019). The majority of nausea and vomiting adverse events were reported as mild to moderate in severity (grades 1 or 2). Severe (grade 3) nausea and/or vomiting occurred in 3 (1.4%) tigecycline-treated and 2 (1.0%) levofloxacin-treated patients. In the tigecycline group, the median time to onset of nausea and/or vomiting was approximately 1.1 days. The total duration of nausea or vomiting occurred over a median of approximately 2.5 days while on tigecycline therapy. Significantly more tigecycline-treated subjects (46; 21.3%) received concomitant medications for nausea/vomiting compared with the levofloxacin-treated subjects (11; 5.2%) (*P *≤ 0.0001). There was no significant difference in the discontinuation rate from study medication between the treatment groups for nausea or vomiting.

**Table 6:** Treatment-Emergent Adverse Events that Occurred in ≥ 3% of Patients (mITT Population), Number of Patients (%)

**Body System**	**Tigecycline**		**Levofloxacin**		**Total**		**Fisher Exact**
**Adverse Event**	**(n = 216)** **N (%)**		**(n = 212)** **n (%)**		**(N = 428)** **N (%)**		***P*-Value**
Any adverse event	135	62.5	100	47.2	235	54.9	0.002*
Body as a whole	29	13.4	26	12.3	55	12.9	0.774
Headache	10	4.6	4	1.9	14	3.3	0.173
Digestive system	82	38.0	44	20.8	126	29.4	<0.001*
Diarrhoea	16	7.4	17	8.0	33	7.7	0.858
Nausea	58	26.9	18	8.5	76	17.8	<0.001*
Vomiting	36	16.7	14	6.6	50	11.7	0.001*
Haemic and lymphatic system	31	14.4	11	5.2	42	9.8	0.002*
Leukocytosis	15	6.9	2	0.9	17	4.0	0.002*
Thrombocythaemia	11	5.1	4	1.9	15	3.5	0.112
Metabolic and nutritional	20	9.3	26	12.3	46	10.7	0.351
ALT/SGPT increased	4	1.9	9	4.2	13	3.0	0.169
AST/SGOT increased	4	1.9	7	3.3	11	2.6	0.378
Hypokalaemia	1	0.5	8	3.8	9	2.1	0.019*

Abbreviation: ALT/SGPT = alanine aminotransferase (serum glutamic pyruvic transaminase); AST/SGOT = aspartate aminotransferase/serum glutamic oxaloacaetic transaminase.

Some subjects had more than 1 treatment-emergent adverse event.

*Significant between-group difference at 0.05 level.

Drug-related adverse events (as assessed by the investigator) were reported in 44.4% of tigecycline- and 29.2% of levofloxacin-treated subjects (*P *< 0.001; Table 7). Drug-related digestive system adverse events were the most frequently reported AEs in both treatment groups (32.4% for tigecycline versus 16.0% for levofloxacin; *P *< 0.001). Nausea and vomiting occurred more commonly in tigecycline-treated subjects (25% and 14.4%, respectively) compared with levofloxacin-treated subjects (7.5% and 4.7%, respectively; both *P *< 0.001).

**Table 7:** Drug-Related Adverse Events that Occurred in ≥ 3% of Patients (mITT Population), Number of Patients (%)

**Body System**	**Tigecycline**		**Levofloxacin**		**Total**		**Fisher Exact**
**Adverse Event**	**(N = 216) ** **n (%)**		**(N = 212) ** **n (%)**		**(N = 428)** ** n (%)**		***P*-Value**
Any adverse event	96	44.4	62	29.2	158	36.9	0.001*
Body as a whole	10	4.6	10	4.7	20	4.7	1.000
Cardiovascular system	9	4.2	5	2.4	14	3.3	0.416
Digestive system	70	32.4	34	16.0	104	24.3	<0.001*
Diarrhoea	15	6.9	14	6.6	29	6.8	1.000
Nausea	54	25.0	16	7.5	70	16.4	<0.001*
Vomiting	31	14.4	10	4.7	41	9.6	<0.001*
Haemic and lymphatic system	7	3.2	4	1.9	11	2.6	0.544
Metabolic and nutritional	15	6.9	16	7.5	31	7.2	0.854

Some subjects had more than 1 drug-related adverse event.

*Significant between-group difference at 0.05 level.

Thirty-nine patients reported a serious adverse event during the study period (18 tigecycline, 21 levofloxacin). Only one serious adverse event (nausea) in a tigecycline-treated patient was considered related to the study drug; the event resolved. A total of twelve (12) patients died during the study: 7 in the tigecycline group and 5 in the levofloxacin treatment group. All of the deaths were reported by investigators as either probably not or definitely not related to the study drug.

There was no statistically significant difference between tigecycline and levofloxacin in the number of patients in the mITT population who prematurely discontinued the study drug (14; 6.5% tigecycline versus 17; 8.0% levofloxacin) or withdrew because of any adverse event (4; 1.9% tigecycline versus 5, 2.4% levofloxacin). Mean change from baseline in laboratory, vital sign, and ECG parameters was generally small.

## Discussion

This multinational, double-blind, randomized, phase 3 clinical trial demonstrated that IV tigecycline (100 mg initial dose, followed by 50 mg every 12 hours) is as effective as IV levofloxacin (500 mg once- or twice-daily) for the treatment of hospitalized adult patients with CAP. For the 280 clinically evaluable patients, clinical cure rates were 88.9% for tigecycline and 85.3% for levofloxacin at the test-of-cure visit, with tigecycline meeting the statistical criteria for non-inferiority compared with levofloxacin, a widely used agent in this setting. These findings were confirmed in the co-primary c-mITT population. We also observed that tigecycline generally achieved good cure rates and was very consistent when analyzed by demographic characteristics and co-morbid conditions, and by a variety of risk factors, including the Fine Pneumonia Severity Index [16]. Because bacteremia can be a fatal complication among patients with CAP, it is also encouraging that tigecycline provided an excellent cure rate (90.9%) in ME patients with *S. pneumoniae *bacteremia.

Because most of the microbiologic eradication rates by patient were presumed based on clinical response, little can be said about this secondary endpoint. ME subjects with *S. pneumoniae *isolates, the most common isolate by far, achieved clinical cures in 92% and 86% of tigecycline- and levofloxacin-treated subjects, respectively. Although the number of penicillin-intermediate or -resistant strains was low, both antimicrobials eradicated 100% of penicillin-intermediate (n = 8) and penicillin-resistant (n = 5) strains, although 1 of the 3 levofloxacin-treated subjects with penicillin-resistant *S. pneumoniae *did not achieve a clinical cure. High clinical cure/eradication rates were achieved against *M. pneumoniae*, the second most commonly identified organism, with both treatments; the cure rates for the ME population were 96% and 92%, respectively. Good cure rates were generally achieved with tigecycline against a number of other commonly encountered respiratory pathogens, including *Legionella pneumophila *(although there were small numbers of subjects with this pathogen), supporting *in vitro *observations that tigecycline has broad-spectrum activity against isolates frequently encountered in patients with CAP [15,18,19].

The current study also confirmed the *in vitro *activity of tigecycline against respiratory isolates, with MIC_90_s of ≤ 1.0 μg/mL against the common gram-positive and gram-negative respiratory aerobes. Tigecycline has good *in vitro *activity against resistant organisms (e.g., penicillin-resistant *S. pneumoniae*,) [15,18,19]. It is now recognized that tigecycline binds to bacterial ribosomes in a novel way that allows it to overcome tetracycline resistance due to ribosomal protection [20].

Tigecycline and levofloxacin were generally well-tolerated in the current trial. Tigecycline-treated patients reported significantly more treatment-emergent adverse events overall (62.5% versus 47.2%; *P *= 0.002), as well as adverse events considered drug-related by the investigator (44.4% versus 29.2%; *P *< 0.001). As has been reported in other tigecycline studies, gastrointestinal adverse events were the most frequently reported treatment-emergent and drug-related adverse events. For treatment-emergent adverse events, this was the case in both the tigecycline (38%) and levofloxacin treatment groups (21%; *P *< 0.001). While rates of nausea and vomiting were reported significantly more often among tigecycline patients, most events occurred early, were of mild to moderate severity, and resolved within a few days without the need to stop the drug prematurely. However, concomitant medications for nausea/vomiting were given four times as often among tigecycline patients (*P *≤ 0.0001). There were no significant differences between treatment groups in the frequency of serious adverse events, discontinuations, including those from adverse events, and deaths. There were 12 deaths in the study (2.8%); none of the deaths were considered by the investigators to be related to the study drugs. The adverse event profile after tigecycline therapy in this study supports previous safety data from phase 3 studies [21,22].

## Conclusion

In summary, IV tigecycline was found to be generally well-tolerated and comparable with respect to efficacy to IV levofloxacin in the treatment of hospitalized adult patients with CAP. Results of this phase 3 study also demonstrate that tigecycline achieved good cure rates against the most frequently encountered respiratory pathogen, *S. pneumoniae*, and against the other common respiratory pathogens, including *Legionella pneumophila *and other atypical organisms. Treatment of CAP is complicated by rising rates of antibiotic-resistant bacteria, and there is growing concern about widespread fluoroquinolone use and rising rates of fluoroquinolone-resistance among *S. pneumoniae*. Based on the results of this study, tigecycline may provide an alternative option for the treatment of hospitalized patients with CAP [23-25].

## Competing interests

Wyeth Research funded this study. ND, NC, HG, and AC are employees of Wyeth Research. The remaining authors declare that they have no competing interests.

## Authors' contributions

CT, SM, PIC, JS, MZ, and VC conducted the study, contributed to data acquisition, and reviewed and approved the draft manuscript. ND, NC, HG, and AC contributed to, reviewed, and approved the draft manuscript. All authors read and approved the final manuscript.

## Pre-publication history

The pre-publication history for this paper can be accessed here:


